# C1QBP promotes apoptosis of goat fetal turbinate cells via inhibiting the expression of TRIM5 or TNFSF10

**DOI:** 10.3389/fvets.2025.1524734

**Published:** 2025-05-16

**Authors:** Qiuyun Li, Zhengding Tang, Hua Xiang, Yixin Dan, Yupeng Ren, Huanrong Zhang, Falong Yang, Jiangjiang Zhu

**Affiliations:** ^1^Key Laboratory of Animal Medicine at Southwest Minzu University of Sichuan Province, Chengdu, China; ^2^Qinghai-Tibetan Plateau Animal Genetic Resource Reservation and Utilization, Key Laboratory of Sichuan Province, Chengdu, China

**Keywords:** C1QBP, apoptosis, RNA-seq, TRIM5, TNFSF10

## Abstract

**Background:**

Infectious ecthyma is a severe and highly contagious disease caused by ORF virus (ORFV). The virus is responsible for significant economic losses in the goat industry and threatens humans. Regarding complement component 1, q subcomponent binding protein (C1QBP), we previously showed that C1QBP can interact with ORFV129. However, the role of C1QBP in regulating the apoptosis of goat fetal turbinate cells is unknown.

**Methods:**

After pcDNA3.1-*C*1*QBP* and siRNA were transfected into goat fetal turbinate cells (GFTCs), the expression of C1QBP was detected by western blot and cell cycle and apoptosis using flow cytometry. The expression of cycle-related genes *cyclin-dependent protein kinase* 2 (*CDK*2) and *cyclin-dependent kinase inhibitors* 1*A* (*p*21) and apoptosis-related genes *cysteinyl aspartate specific proteinase* 3 (*Caspase* 3), *cysteinyl aspartate specific proteinase* 7 (Caspase 7), *p*53, *poly* (*ADP-ribose*) *polymerase* 1 (*PARP*1), *B-Cell lymphoma*-2 *Like*-11 (*BCL*2*L*11), *B-cell lymphoma* 2 *associated X protein* (*Bax*), and *B-cell lymphoma* 2 (*Bcl*-2) were tested by real-time quantitative PCR (RT-qPCR). The effect of MTT on the proliferation of GFTCs was detected. The localization of C1QBP in GFTCs was detected by inverted fluorescence microscope. Finally, transcriptome sequencing was performed and validated by siRNA treatment knockdown of C1QBP, and screening two genes tripartite motif containing 5 (*TRIM*5) and tumor necrosis factor superfamilymember10 (*TNFSF*10) with significant changes in expression levels and relevance to cell apoptosis, and to verify their roles in C1QBP-induced cell apoptosis.

**Results:**

Knockdown of C1QBP significantly increased cell viability; cells remained in the G0/G1 phase and reduced apoptosis. Knockdown of C1QBP reduced the mRNA expression of *CDK*2, *p*21, *Caspase*3, *Caspase*7, *Bax, PARP*1, *BCL*2*L*11, and *p*53, up regulated the mRNA expression of the *Bcl*-2. Except for *Bcl*-2, The opposite effect was observed when C1QBP was overexpressed in GFTCs and the mRNA levels of *Bcl*-2 had no significant effect. Immunostaining revealed intracellular localization of C1QBP, primarily in the cytoplasm of the GFTCs. Furthermore, gene expression profiling analysis in C1QBP depleted cells compared to the control revealed that a total of 236 differential expression genes (DEGs), including 119 up regulated DEGs and 117 down regulated DEGs, and the expression of *TRIM*5 and *TNFSF*10 genes were significantly upregulated. Pathway analysis were predicted to be enriched in Herpes simplex virus 1 infection, Pathways in cancer, PI3K-Akt signaling pathway, Apoptosis, Apoptosis-multiple species and p53 signaling pathway. C1QBP reduced the expression of *TRIM*5 and *TNFSF*10 genes. Knockdown of TRIM5 promoted apoptosis in GFTCs, but silencing of *TNFSF*10 had no change in apoptosis rate. In particular, the apoptosis rate was significantly increased in the TRIM5-siRNA2 or TNFSF10-siRNA2 and C1QBP-siRNA2 group compared to the only C1QBP-siRNA2 group.

**Conclusion:**

These findings provide a deeper understanding of the role of C1QBP in apoptosis and could pave the way for further study investigating the role and mechanism of C1QBP protein in mediating the regulation of cell apoptosis by ORFV.

## Highlights

- Knockdown of C1QBP by siRNA treatment increased cell viability, cells remained in G0/G1 phase, and reduced cell apoptosis. Whereas, the opposite effect was observed when C1QBP was overexpressed.
- The intracellular localization of C1QBP occurred mainly in the cytoplasm of GFTCs.
- Gene expression profiling and pathway analysis in C1QBP depleted cells compared to the control revealed that a total of 236 differential expression genes (DEGs), including 119 upregulated DEGs and 117 downregulated DEGs, and were predicted to enrich in Herpes simplex virus 1 infection, Pathways in cancer, PI3K-Akt signaling pathway, Apoptosis, Apoptosis-multiple species and p53 signaling pathway.
- The role of C1QBP in cell apoptosis could be regulated by the expression of TRIM5 or TNFSF10.

## 1 Introduction

Infectious ecthyma (Orf) is a zoonotic infectious disease caused by goat's aphthous virus (ORFV) infection. This virus mainly infects sheep and goats, especially lambs. Humans can also be infected through contact with virus-laden blood and tissues (1). The incidence rate of ORFV infection in newborn and young lambs can reach up to 100%, with a mortality rate of 93%. Although the mortality rate is relatively lower in adult goat infected with ORFV, it can still lead to weight loss, slow growth, and secondary infections. The vaccines currently available on the market cannot provide full immune protection, and there is no specific treatment (2). In recent years, with the increase in goat farming and cross-border circulation in China, ORFV causes considerable economic losses in the agriculture industry and threatens human health. ORFV is a double-stranded DNA virus in the genus *Parapoxvirus* (3). The ORFV genome comprises a central genomic region (ORFVs 001–008 and ORFVs 112–134) and a terminal genomic region (ORFVs 009–111). Ankyrin-repeat (ANK) protein, as a virulence factor encoded by ORFV, plays a significant role in mediating viral infection. Firstly, Ankyrin-repeat (ANK) protein can activate the ubiquitin-dependent programmed degradation of cellular proteins by interacting with cellular ubiquitin ligases, thereby regulating the cellular response to viral infection (4). Secondly, Ankyrin-repeat (ANK) protein can regulate the activity of the NF-κB signaling pathway by interacting with NF-κB pathway proteins, and thereby trigger viral immune evasion (5). Finally, the ankyrin-repeat (ANK) protein of poxviruses can regulate the survival and replication of the virus within cells by binding to host proteins (6). The terminal regions of the ORFV genomes are responsible for encoding five ankyrin-repeat (ANK) proteins, namely ORFV008, ORFV123, ORFV126, ORFV128, and ORFV129 (7). However, there is currently limited research on the functions of these ORFV ankyrin proteins. ORFV129 has six ANKs at its N-terminal end and one F-box-like domain at the C-terminal (8). Our previous study showed that ORFV129 can interact with proteins such as complement component 1, q subcomponent binding protein (C1QBP), and overexpression of C1QBP played a cellular antiviral role during ORFV infection, while C1QBP knockdown can facilitate the replication of the ORFV viral genome and the production of progeny viruses (9). This suggests that goat C1QBP has a regulatory role in the replication of ORFV. However, the specific mechanism by which C1QBP regulates ORFV replication remains unclear.

Complement 1q binding protein (C1QBP, also known as p32 or globular C1q binding protein or HABP1) was initially thought to be a splicing factor of intracellular pre-mRNA, interacting with the intracellular splicing factor ASF/SF2 (splicing factor-2) and regulating the function of SF2 (10, 11). Subsequently, C1QBP was found to be a counterpart of the yeast p30 gene, and it was shown that C1QBP and p30 proteins were localized in the mitochondrial matrix and were necessary for the maintenance of mitochondrial oxidative phosphorylation (12). In 2004, C1QBP was found to interact with platelets to regulate and repair vascular injury and inflammation (13, 14). C1QBP is one of the receptors of C1q, which combines with the spherical head of C1q to form the complement system in the body (15). The activation of the complement system regulates the defense function of the host immune system and produces a series of biological responses (16). Over time, C1QBP was found to be involved in the regulation of cellular immunity, metabolism, proliferation, apoptosis, and other biological processes (17–19). For example, C1QBP regulates the immune response by binding to the globular head domain of C1q. C1QBP improves vascular permeability by up regulating the expression of bradykinin receptor 1 (20). The purified porcine C1QBP protein increased mRNA levels of LPS/PRRSV (Porcine Reproductive and Respiratory Syndrome Virus)-induced inflammatory factors such as IL-1β, IL-6, IL-8, TNF-α, and RANTES. Also, it enhanced PRRSV-induced NF-κB activation and p65 phosphorylation, inducing inflammatory responses (21). C1QBP regulates the secretion of cytokines IL-6, IL-1β, and IFN-γ via interaction with ORFV129 (9). C1QBP expression of *Larimichthys crocea* was increased upon infection with *Pseudomonas polyglossia*, which caused the immune response to visceral leukoplakia (22). Meanwhile, C1QBP also plays a significant role in regulating cell apoptosis. Such as, C1QBP was reported to induce mitochondrial dysfunction, growth retardation, and apoptosis when stably overexpressed in normal fibroblasts (23). The high levels of expression of RPAIN in preeclampsia may affect the biological function such as proliferation, apoptosis, and invasion of placental trophoblast cells through C1QBP, which participates in the occurrence and development of preeclampsia (24). C1QBP promoted ROS production, apoptosis, and the expression of Bax and active Caspase 3 (25).

ORFV infection can induce cell apoptosis (26), and the ORFV129 protein is an ankyrin-repeat (ANK) protein that plays a significant role during viral infection. Based on our previous findings, there is an interaction between ORFV129 and C1QBP. Given that C1QBP is a gene related to cell apoptosis, we speculate that C1QBP may be involved in the phenomenon of cell apoptosis caused by ORFV infection. However, the specific mechanism of C1QBP in regulating cell cycle and apoptosis is unclear. This has also seriously affected the discussion on the role of C1QBP in regulating cell apoptosis caused by ORFV infection. The goat host protein C1QBP as the material in the present study. We examined the role of C1QBP in the cell cycle, apoptosis, and proliferation by RNA silencing and over expression. In addition, RNA-seq was performed to identify differential transcripts affected by C1QBP loss of function and to identify potential genes and signaling pathways that could mediate C1QBP-associated apoptosis.

## 2 Materials and methods

### 2.1 Animals and cell culture

A 2-day-old male Jianzhou big-eared goat was purchased from Dagda Animal Husbandry Co., Ltd. (Jianyang City, Sichuan Province, China). Animal care and experiments were performed according to the regulations of the Administration of Affairs Concerning Experimental Animals (Ministry of Science and Technology, China, revised in June 2004), and all experimental procedures were reviewed and approved by the Institutional Animal Care and Use Committee, Southwest Minzu University (Chengdu, Sichuan, China).

The primary culture of goat fetal turbinate cells (GFTCs) was carried out according to Dan et al. (9) and was preserved in our laboratory. Briefly, a 2-day-old goat was fed freely in the house with milk and performed well during experiments. The turbinate bone tissues were collected after slaughter and immediately frozen in liquid nitrogen until it was transported back to the laboratory. In a laminar flow cabinet, the turbinate bone tissues were washed several times with phosphate-buffered saline. The smaller pieces of tissue blocks were maintained in Dulbecco's minimal essential medium (Hyclone, Beijing, China) containing 15% fetal bovine serum and supplemented with 10 kU/L penicillin and streptomycin (Boster, Wuhan, China) in 6-well plates for cell culture in a 5% CO_2_ incubator (Thermo Fisher Scientific, Waltham, MA, USA) at 37°C, and the medium changed after 48 h. The primary GFTCs reached ~80% confluence and were passaged at a 1:2 ratio using 0.25% trypsin.

### 2.2 Plasmid constructs and chemical synthesis of siRNA

These expression vectors pcDNA3.1-*C*1*QBP* and C1QBP-siRNA2, which have been shown to silence C1QBP expression in GFTCs, were constructed and conserved in our laboratory (9, 27). The siRNAs were designed and synthesized using the goat gene *TRIM*5 (JX649920.1) and predicted gene *TNFSF*10 (XM_005675308.3) sequence as a target by Shanghai GenePharma Co., Ltd. Three specific siRNA pairs were designed as follows Table 1.

**Table 1:** TNFSF10 and TRIM5 interfering fragment sequences.

**Sequence name**	**Sequence (5′-3′)**
TNFSF10-siRNA1	F:GAGGGUAGCUGCUCAUAUATT
	R:UAUAUGAGCAGCUACCCUCTT
TNFSF10-siRNA2	F:GGGAACUGUUUCAACAGAATT
	R:UUCUGUUGAAACAGUUCCCTT
TNFSF10-siRNA3	F:GGAGGAAUAUUUGAGCUUATT
	R:UAAGCUCAAAUAUUCCUCCTT
TRIM5-siRNA1	F:GCCUGUAUCACUGCAAACATT
	R:UGUUUGCAGUGAUACAGGCTT
TRIM5-siRNA2	F:GCUGAGGAGUUAGAAAUUATT
	R:UAAUUUCUAACUCCUCAGCTT
TRIM5-siRNA3	F:CCAGUUACCAAUGCUAUAATT
	R:UUAUAGCAUUGGUAACUGGTT
Negative control	F:UUCUCCGAACGUGUCACGUTT
	R:ACGUGACACGUUCGGAGAATT

### 2.3 Goat fetal turbinate cells transfection

GFTCs from passage six were used for subsequent experiments, and the experimental treatment was performed when the GFTCs reached 70–80% confluence. The transient transfection cells were treated with Lipofectamine3000 transfection reagent (Thermo Fisher Scientific, Waltham, MA, USA). Cells were collected after 48 h of transfection. Total RNA was extracted using trizol reagent (Takara, Dalian, China), and 1 μg of total RNA was reverse transcribed using a Revert Aid First Strand cDNA Synthesis Kit (Thermo, USA) according to the manufacturer's protocol.

### 2.4 Real-time quantitative PCR (RT-qPCR)

RT-qPCR assays for related gene expression were performed using the SYBR Green kit. The ubiquitously expressed transcript gene was used as an internal reference gene, and the relative expression was calculated using the 2^−ΔΔCt^ method. The total volume of RT-qPCR was 20 μL: 10 μL SYBR^®^ Premix Ex Taq TM (Takara, Dalian, China), 1 μL template cDNA, 1 μL upstream primer, 1 μL downstream primer, and 7 μL ddH_2_O. The PCR conditions included an initial step at 95°C for 3 min and 95°C for 10 s, followed by 40 cycles of amplification and quantification (60°C for 30 s, 72°C for 30 s). The primer information for RT-qPCR information is listed in Table 2.

**Table 2:** Information about primers sequences of genes in goat.

**Name of gene**	**Primer sequence (5′-3′)**	**Tm/°C**	**Use**	**Prouducts length/bp**
*p*53	F:AGTGTGGTGGTGCCCTATGAGTC	60°C	RT-qPCR	137
	R:GAGTCTTCCAGTGTGATGATGGTGAG			
*Caspase*3	F:GGACUGCCUUUACCAACAATT	60°C	RT-qPCR	82
	R:UUGUUGGUAAAGGCAGUCCTT			
*Caspase*7	F:UUCUCCGAACGUGUCACGUTT	60°C	RT-qPCR	101
	R:ACGUGACACGUUCGGAGAATT			
*Bcl*-2	F:TGTGGATGACCGAGTACCTGAACC	60°C	RT-qPCR	127
	R:AGAGACAGCCAGGAGAAATCAAACAG			
*Bax*	F:CGCATTGGAGATGAATTGGACAGTAAC	60°C	RT-qPCR	128
	R:CAGTTGAAGTTGCCGTCGGAAAAC			
*BCL*2*L*11	F:AACCTTCCGATGTAAGTTCTGAGTGTG	60°C	RT-qPCR	117
	R:TGTCTTGCCGCTCTGTCTGTAAAG			
*PARP*1	F:CCAACATCCGTGTCGTGTCTGAG	60°C	RT-qPCR	102
	R:CCACTGCTTCAACAGGCTCCAC			
*CDK*2	F:CACCTCCACACCATCCACAGTAATAC	60°C	RT-qPCR	140
	R:GCACTCACTGGCATTCCTCTTCC			
*P*21	F:GGTAGAGTTGGCTTTATGGGACACAG	60°C	RT-qPCR	114
	R:TCAGGGCTATCAATGGAGAAACACATC			
*UXT*	F:GCAAGTGGATTTGGGCTGTAAC	60°C	RT-qPCR	180
	R:ATGGAGTCCTTGGTGAGGTTGT			
*VGF*	F:TCGGTCATGAAATCGCTCAG	58.1°C	RT-qPCR	143
	R:CCAGCTACCGGCTCTTTATG			
*TNFSF*10	F:CAAACACATGGTGGAAGTAAGG	57.56°C	RT-qPCR	106
	R:CAGGGAGCTGAGTGGTAAAG			
*PLAC*8	F:ACCCTCTACAGGACTCGATAC	57.7°C	RT-qPCR	125
	R:CGATTGGCTCTCCTTCTGTT			
*LOC*102169889	F:CTTCTTACACTCACACCCACTC	58.01°C	RT-qPCR	97
	R:CAAGGCCAACCCGTAAGAA			
*LOC*102186893	F:GGTATCCTTGTCGTCTTCTTCC	58.02°C	RT-qPCR	110
	R:GTGGACTCACAGTGCTTCAT			
*ISG*20	F:TGCTGTACGACAAGTTCATCC	58.15°C	RT-qPCR	82
	R:ATGTTCCGAGCTGTGATTCC			
*LOC*102173932	F:GACAGCGACTAACCAGTATCAG	58.14°C	RT-qPCR	131
	R:GACCTTGGTGACCTTGATGTAA			
*TNNI*1	F:CAGGGAGATCAAAGACCTGAAG	58.04°C	RT-qPCR	136
	R:CATAGACACCTTGTGCTTGGA			
*NDUFA*4*L*2	F:GCAGTTTCCACTGACTACAAGA	58.34°C	RT-qPCR	119
	R:GGTGGAGAGTGGAAGAAATGAG			
*SCUBE*3	F:CCATCCTCCATTACCACCTATG	57.69°C	RT-qPCR	110
	R:CTGTTGGCTTCACTTGTCTTG			
*HMCES*	F:AAGGTCTGGGACAACTGGA	58.25°C	RT-qPCR	104
	R:CCGTGATGATGCTGTAGGAATAG			
*NCAM*1	F:CAGATGGGAGAGGATGGAAAC	58.01°C	RT-qPCR	94
	R:CTCGGTATTTGACCAGGTAGTG			
*RASD*1	F:CTTACCGGAGACGTGTTCATC	58.20°C	RT-qPCR	101
	R:CACGACTTGGTGTCGAGAAT			

### 2.5 Western blot

SDS-PAGE was performed using a 10-well gel, and sample volumes were 20 μg per well. The electrophoresis conditions were 80 V, 50 min; 120 V, 30 min. After SDS-PAGE electrophoresis, the total protein of the cells was transferred to polyvinylidene fluoride (PVDF) membranes using a semi-dry transfer printer and the transfer printer conditions were 12 V, 20 min, using 5%BSA blocked for 2 h. Mouse monoclonal Flag antibody (Boster, Wuhan, China, M30971-2) and rabbit beta Actin antibody (zenbio, Chengdu, China, T200068-8F10) were then added and incubated overnight at 4°C. Goat anti-mouse IgG (Boster, Wuhan, China, BA1050) and goat anti-rabbit IgG (Boster, Wuhan, China) were labeled with HRP and incubated for 2 h on a room-temperature shaker, followed by imaging using ECL reagent (4Ablotech, Suzhou, China).

### 2.6 Immunofluorescence localization analysis

GFTCs were transfected with pcDNA3.1-*C*1*QBP* and empty vectors pcDNA3.1(+) as control. After a 48 h incubation, the cells were fixed with formaldehyde for 15 min, blocked with 5% BSA for 1 h at 37°C. Mouse monoclonal Flag antibody (Boster, Wuhan, China, M30971-2) were then added and incubated overnight at 4°C. Goat anti-mouse IgG (Boster, Wuhan, China, BA1101) were labeled with FITC and incubated for 2 h in the dark on a room-temperature shaker, and with DAPI (Boster, Wuhan, China) in the dark for 15 min. Then, cells were observed under an inverted fluorescence microscope (Olympus, Japan).

### 2.7 MTT for goat fetal turbinate cells proliferation analysis

The MTT assay was used to evaluate GFTC proliferation. The sixth-generation GFTCs were inoculated in 96-well plates (LABSELECT, Anhui, China) and transfected after 24, 48, 72, and 96 h of treatment with 10 μL of MTT solution (Boster, Wuhan, China) for 4 h at 37°C and the addition of 100 μL of dimethyl sulfoxide (Boster, Wuhan, China) per well. The plates were left for 10 min and incubated for long periods. Finally, the OD of each well was measured using a microplate reader at a wavelength of 490 nm. Five biological replicates were performed, and the experiment was repeated three times.

### 2.8 Goat fetal turbinate cells cycle and cycle-related gene analysis

The sixth-generation GFTCs inoculated into twelve-well plates (LABSELECT, Anhui, China) were transfected using the same method as for the MTT assay. After 48 h of transfection, samples were collected according to the steps of the cell cycle detection kit (Multi Sciences, Hangzhou, China). Cell cycle analysis was then performed using flow cytometry. In addition, RT-qPCR was used to analyze the effects of the cycle-related genes *CDK*2 and *p*21. The primer information for RT-qPCR is listed in Table 2. Three biological replicates were performed, and the experiment was repeated three times.

### 2.9 Goat fetal turbinate cells apoptosis and apoptosis-related gene analysis

Sixth-generation GFTCs inoculated in 12-well plates were transfected using the same method as the cell cycle assay. After 48 h of transfection, samples were collected according to the instructions of the PI Apoptosis Kit (MultiSciences, Hangzhou, China). The effects of apoptosis-related genes *p*53, *Caspase*7, *Caspase*3, *Bcl*-2, *Bax, BCL*2*L*11, and *PARP*1 were analyzed by RT-qPCR. Information about RT-qPCR primers information were listed in Table 2. Three biological replicates were performed, and the experiment was repeated three times.

### 2.10 RNA sequencing (RNA-seq)

The total RNA of each sample (Negative Control and C1QBP knockdown) was extracted with trizol reagent. RNA purity, quantification, and integrity were assessed, and RNA quantification, library preparation, clustering, and sequencing were performed at Oebiotech. The libraries were sequenced on a Lumina Novaseq 6000 platform, generating 150 bp paired-end reads. Approximate raw data was generated for each sample. Raw reads in Fast Quality (Fastq) format were first processed using Fastp, and the low-quality reads were removed to obtain clean reads. Approximately clean readings for each sample were then retained for subsequent analysis. The clean reads were mapped to the reference genome using HISAT2. The FPKM of each gene was calculated, and the read counts of each gene were obtained using HT Seq counting. PCA analyses were performed using R (version 3.2.0) to assess the biological duplication of samples. Differential expression analysis was performed using DESeq2.A *q* value < 0.05 and fold-change >1.5 or < 0.5 were set as the threshold for significant DEGs. The radar map of the top 30 genes was constructed to show the expression of upregulated or downregulated DEGs using R packetage Gradar. Based on the hypergeometric distribution, GO, KEGG Pathway, Reactome, and Wiki pathways enrichment analyses of DEGs were performed to determine the significantly enriched term using R (version 3.2.0). Then, differentially expressed genes based on transcriptome data were selected to check the accuracy of the experimental transcriptome data by RT-qPCR. we used correlation screens between DEGs to analyze genes that may play a key role in the regulation of apoptosis. Finally, the role of apoptosis-related genes *TRIM*5 and *TNFSF*10 in C1QBP promoting apoptosis were studied using flow cytometry.

### 2.11 Statistical analysis

The expression level of C1QBP and cycle-related or apoptosis-related gene data from RT-qPCR in goat tissues were processed with the 2^−ΔΔCt^ method. The ubiquitously expressed transcript was used as an internal control primer. One-way analysis of variance was performed using SPSS 26.0 software, followed by Duncan's multiple comparison test; *p* < 0.05 and *p* < 0.01 represent significant and highly significant differences, respectively. Spearman correlation analysis is used to analyze the correlation between indicators. All experiments were repeated three times.

## 3 Results

### 3.1 Knockdown of C1QBP inhibits apoptosis in goat turbinate bone cells

**Figure 1:**
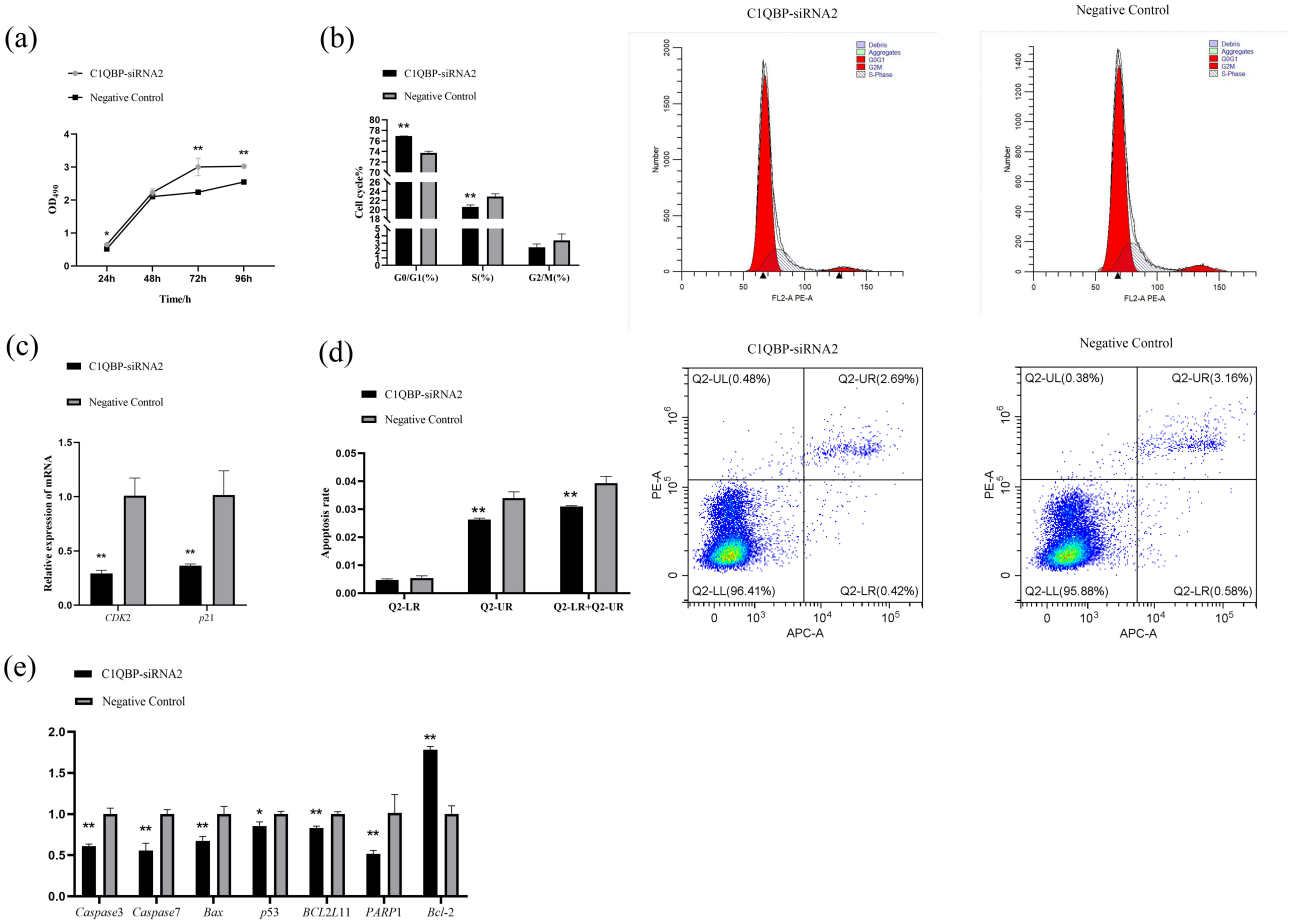
Effect of *C*1*QBP* reduction on the proliferation, cycle, and apoptosis of GFTCs. **(A)** Knockdown of C1QBP promotes the cell proliferation of GFTCs; OD 490 nm characterizes cell viability. **(B)** Effect of *C*1*QBP* deficiency on the cycle in GFTCs. **(C)** Effect of *C*1*QBP* deficiency on the expression of cycle-related genes in GFTCs. **(D)** Effect of *C*1*QBP* deficiency on the apoptosis in GFTCs. **(E)** Effect of *C*1*QBP* deficiency on expression of apoptosis-related genes in GFTCs. ^*^represents 0.01 < *p* < 0.05, ^**^represents *p* < 0.01.

MTT assays were used to evaluate the proliferation and the results showed a significant increase in cell viability (OD 490 nm) in the knockdown C1QBP group compared to the negative control group (*p* < 0.05), and the promotion increased with time (Figure 1A). Flow cytometry was used to demonstrate the function of C1QBP in goat turbinate bone cell cycle. Flow cytometry revealed that 76.9 ± 0.021% of GFTCs represented progressed through G0/G1 when C1QBP knockdown, which was much higher than when the cells were transfected with negative control group. The G2/M+S phase of cells treated with C1QBP siRNA was lower than that of the control group (Figure 1B). Thus, a G1 phase arrest was observed in cells when C1QBP knockdown. In our study, the mRNA expression levels of cell cycle-related genes were examined, and the results showed that knockdown of C1QBP significantly reduced the mRNA levels of *CDK*2 and *p*21 (*p* < 0.01; Figure 1C).

We also examined the responses of apoptosis and apoptosis-related genes to the transfection with C1QBP-siRNA2, as compared to the negative control group. As a result, the knockdown of C1QBP inhibited apoptosis in goat turbinate bone cells (*p* < 0.01; Figure 1D). The mRNA expression levels of the apoptosis-related genes were examined. The results showed that disruption of the expression of the *C*1*QBP* gene significantly downregulated the mRNA expression of the genes *Caspase*3, *Caspase*7, *Bax, PARP*1, and *BCL*2*L*11 (*p* < 0.01), down regulated the mRNA expression of the *p*53 gene (*p* < 0.05), and significantly up regulated the mRNA expression of the *Bcl*-2 gene (*p* < 0.01; Figure 1E).

### 3.2 Over expression of C1QBP promotes apoptosis in goat turbinate bone cells

**Figure 2:**
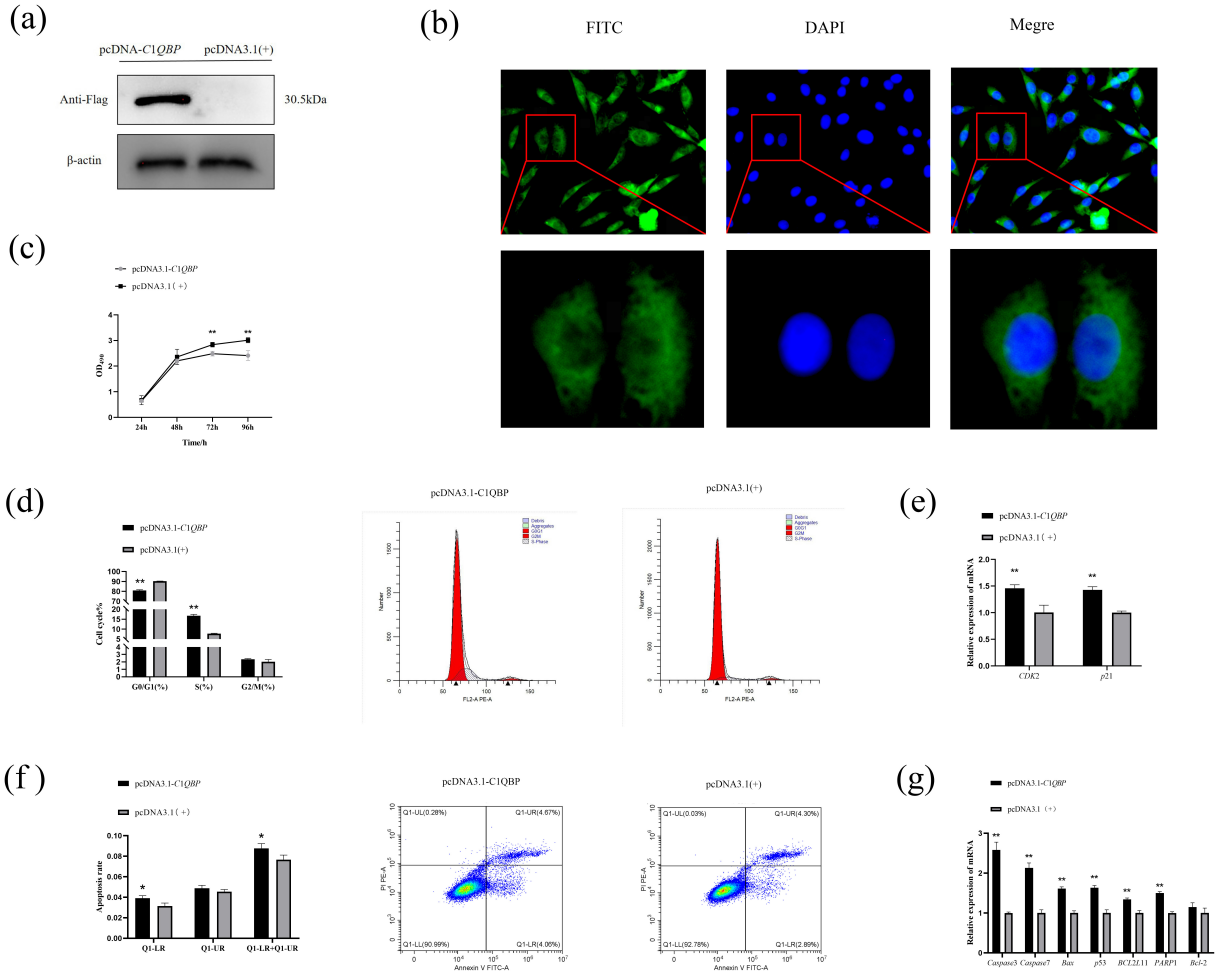
Effect of over expression of *C*1*QBP* on the proliferation, cycle, and apoptosis of GFTCs and Protein expression and subcellular localization of *C*1*QBP*. **(A)** Expression of *C*1*QBP* in GFTCs. **(B)** Localization of *C*1*QBP* in GFTCs. **(C)** Over expression of *C*1*QBP* inhibits cell proliferation in GFTCs. **(D)** Effect of *C*1*QBP* over expression on the cycle in GFTCs. **(E)** Effect of *C*1*QBP* over expression on the expression of cycle-related genes in GFTCs. **(F)** Effect of *C*1*QBP* over expression on the apoptosis in GFTCs. **(G)** Effect of *C*1*QBP* over expression on expression of apoptosis-related genes in GFTCs. ^*^represents 0.01 < *p* < 0.05, ^**^represents *p* < 0.01.

Based on the results that silencing of C1QBP increases cell proliferation, to verify whether over expression of C1QBP could inhibit cell viability. Using β-actin as an internal reference gene, western blotting results showed that the tag protein Flag (30.5 ku) expression was detected in the cells transfected with the recombinant plasmid pcDNA3.1-*C*1*QBP*. In contrast, the cells transfected with empty pcDNA3.1 (+) were detected (Figure 2A). Immunostaining revealed intracellular localization of C1QBP primarily in the cytoplasm of the GFTCs with red light (Figure 2B).

MTT assay was also performed with cells over expressing pcDNA3.1-*C*1*QBP* or pcDNA3.1(+). The results showed that over expression of C1QBP resulted in a significant decrease in cell growth compared to the negative control (Figure 2C). Flow cytometry showed that goat turbinate bone cells were in the G2/M+S phase when C1QBP was over expressed, significantly higher than the control group. The G0/G1phase of cells treated with C1QBP overexpression was lower than that of the pcDNA3.1(+) group (Figure 2D). The mRNA expression levels of *CDK*2 and *p*21 genes were increased after over expression of C1QBP (*p* < 0.01; Figure 2E). C1QBP-overexpressing cells were more susceptible to apoptosis than those expressing pcDNA3.1(+) (Figure 2F). In addition, C1QBP over expression could significantly up regulate the mRNA expression of *Caspase*3, *Caspase*7, *p*53, *PARP*1, *BCL*2*L*11, and *Bax* gene (*p* < 0.01), but had no significant effect on *Bcl*-2 (Figure 2G).

### 3.3 Silencing of C1QBP changes cellular gene expression profile in goat turbinate bone cells

**Figure 3:**
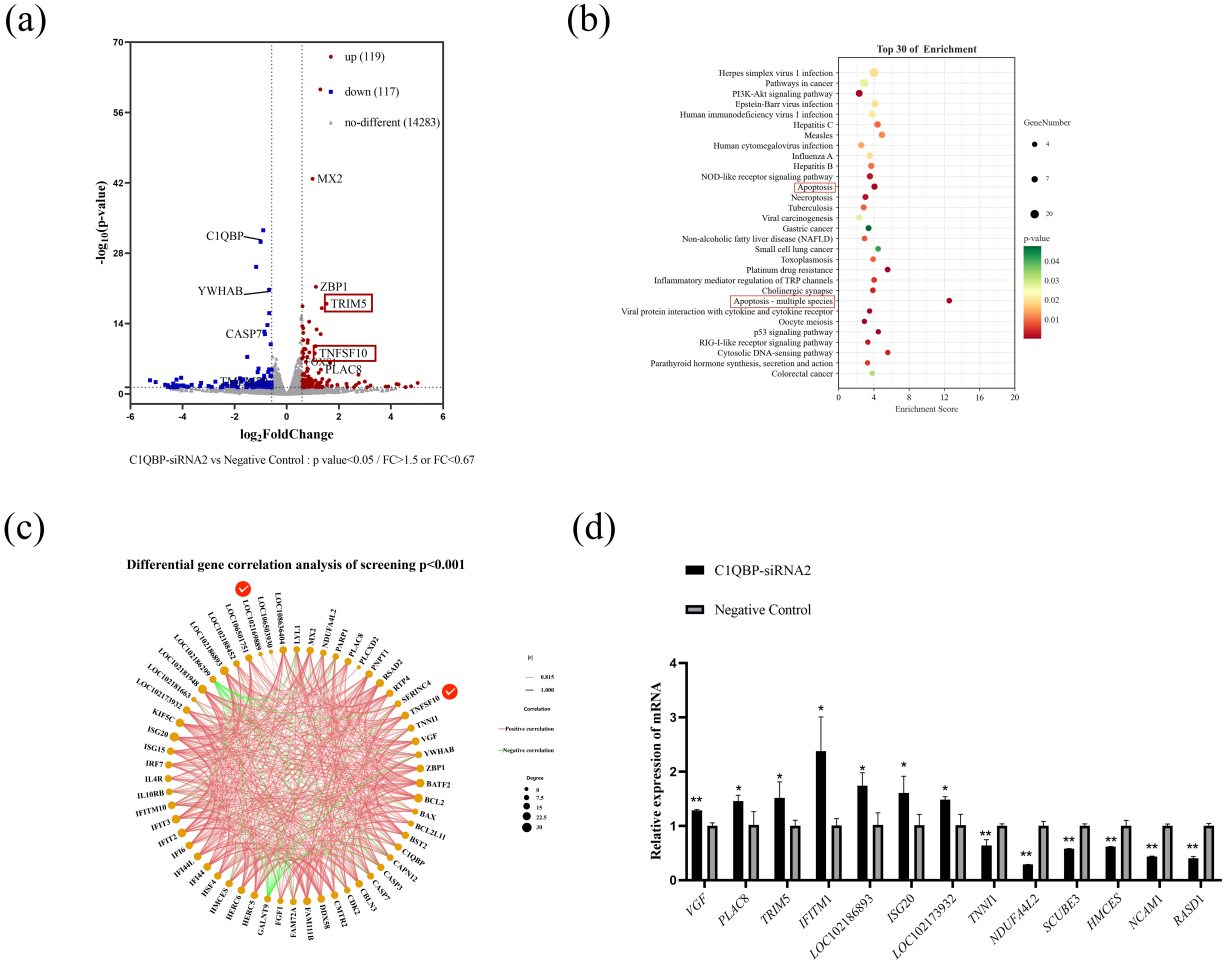
DEGs analysis after C1QBP knockdown. **(A)** Differential representation of volcano diagrams. **(B)** The top 30 enriched pathways of DEGs. **(C)** Visual network diagram for correlation analysis of differentially expressed gene expression **(D)**. RT-qPCR verification of some differential genes. ^*^represents 0.01 < *p* < 0.05, ^**^represents *p* < 0.01.

To investigate the mechanism of action of C1QBP in increasing apoptosis, we sequenced and analyzed the transcriptome of total RNA from C1QBP knockdown and negative controls in goat turbinate bone cells. Principal component analysis showed a significant separation between the interference C1QBP and negative control groups (Supplementary Figure S1). A total of 236 differentially expressed genes (DEGs) were screened out on the volcano map (Figure 3A; Supplementary Table S1) (*p* < 0.05, multiplex change >1.5), of which 119 were up regulated, and 117 were down regulated. In the GO database, DEGs were divided into three directories (Supplementary Figure S3; Supplementary Table S2): molecular functions (MF, 237), cellular components (CC, 175), and biological processes (BP, 666). Go enrichment analysis showed that DEGs were enriched by the response to the virus, activation of the innate immune response, negative regulation of the viral genome, and defense response to the virus.

The figure shows the first 30 significantly enriched KEGG signaling pathways, such as pathways in Herpes simplex virus 1 infection, Pathways in cancer, PI3K-Akt signaling pathway, Apoptosis, Apoptosis-multiple species and p53 signaling pathway (Figure 3B; Supplementary Table S3). Furthermore, we used correlation screens between DEGs to analyze genes that may play critical roles in regulating apoptosis (Figure 3C; Supplementary Table S4). The results showed that the essential genes may be *YWHAB, TNFSF*10, *LOC*102169889 (*TRIM*5), *VGF*, and *PARP*1 etc. In addition, we randomly selected eight up regulated genes *VGF, TNFSF*10, *PLAC*8, *TRIM*5, *LOC*106501751 (*IFITM*1), *LOC*102186893, *ISG*20, *LOC*102173932 (*p* < 0.05, FC > 2, FPKM > 3, FPKM < 50) and seven down regulated genes *TNNI*1, *RPS*25, *NDUFA*4*L*2, *SCUBE*3, *HMCES, NCAM*1, *RASD*1 (*p* < 0.05, FC < 0.67, FPKM > 2) of RNA-seq results for reliability analysis. The results showed that RT-qPCR trends generally agreed well with the RNA-seq expression profiles of randomly selected genes (Figure 3D).

### 3.4 Verification of signaling pathways

**Figure 4:**
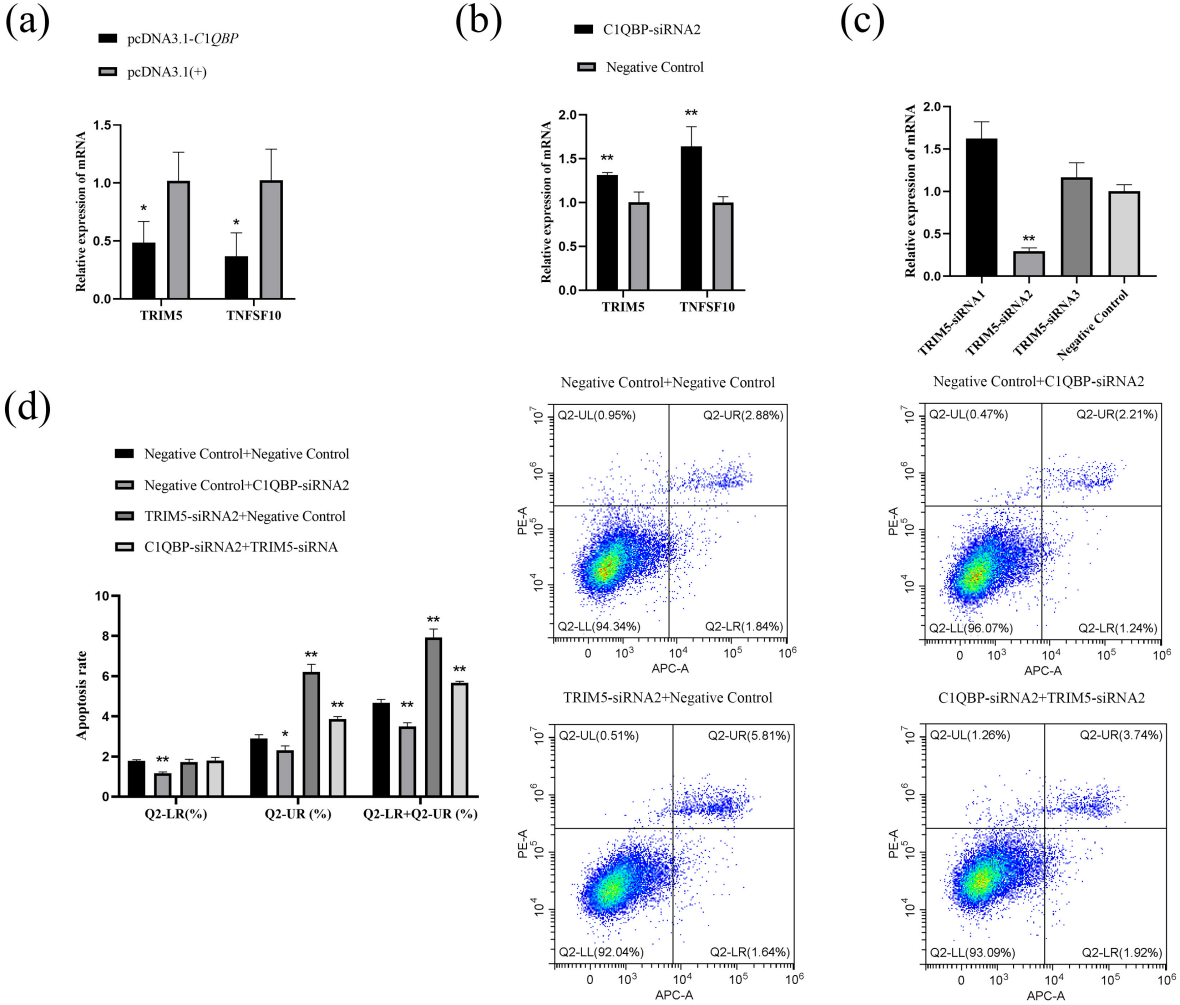
*C*1*QBP* deficiency inhibits apoptosis by regulating *TRIM*5 expression in GFTCs. **(A)** Effect of over expression of C1QBP on TNFSF10 and TRIM5. **(B)** Effect of knockdown of C1QBP on TNFSF10 and TRIM5. **(C)** Screening of interfering fragments for TRIM5. **(D)** Effect of *TRIM*5 and C1QBP on apoptosis of GFTCs. ^*^represents 0.01 < *p* < 0.05, ^**^represents *p* < 0.01.

**Figure 5:**
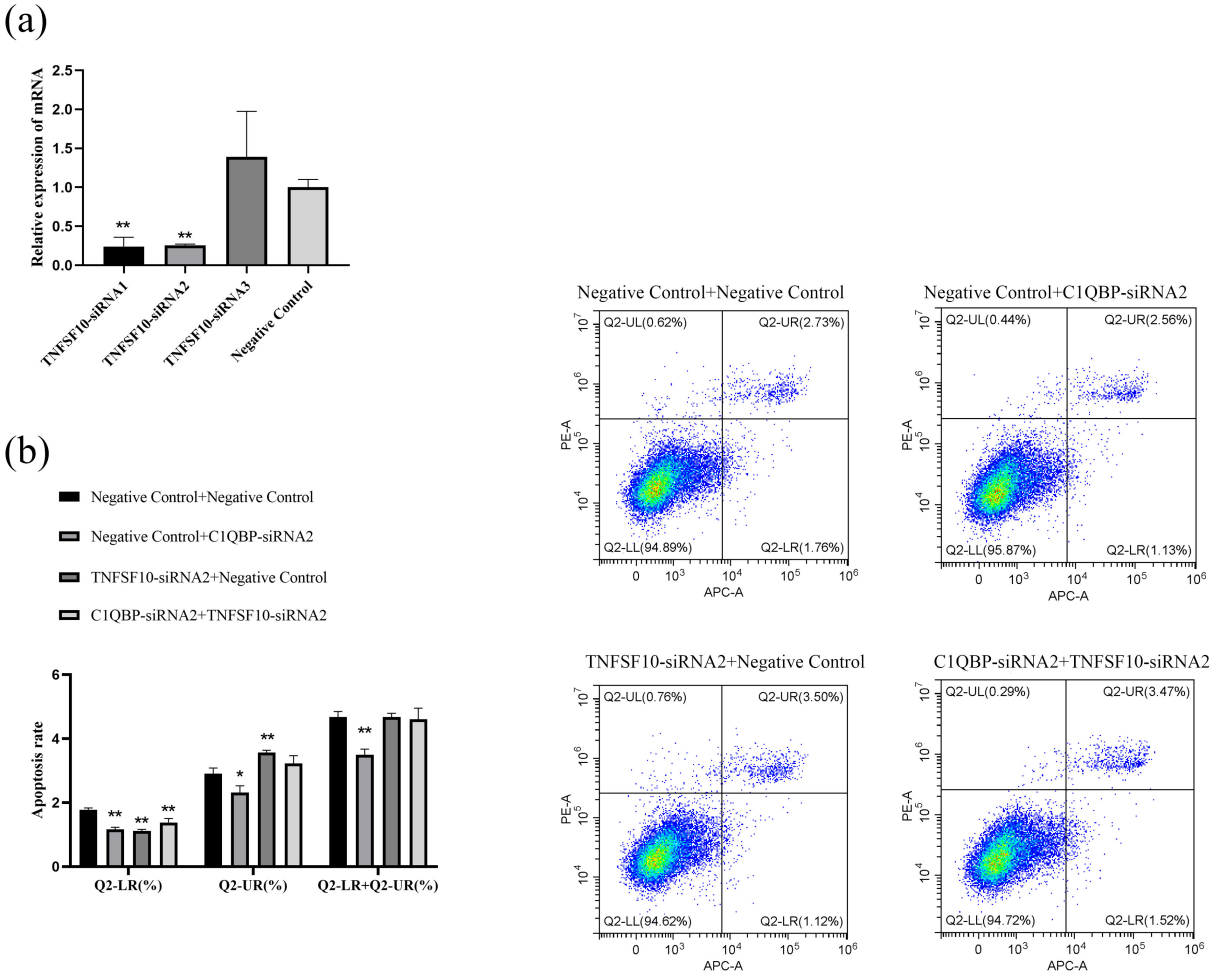
*C*1*QBP* deficiency inhibits apoptosis by regulating *TNFSF*10 expression in GFTCs. **(A)** Screening of interfering fragments for TNFSF10. **(B)** Effect of TNFSF10 and C1QBP on apoptosis of goat turbinate bone cells. ^*^represents 0.01 < *p* < 0.05, ^**^represents *p* < 0.01.

RNA-seq analysis showed that the expression of TRIM5 and TNFSF10 was enriched in the analysis of DEGs. To examine whether C1QBP regulate the expression of TNFSF10 and TRIM5, the pcDNA3.1-*C*1*QBP* or pcDNA3.1(+) was transfected in GFTCs. After 48 h, the cells were collected was analyzed by RT-qPCR. Compared with pcDNA3.1(+) cells, TNFSF10 and TRIM5 mRNA was reduced in pcDNA3.1-*C*1*QBP* cells (Figure 4A). And the opposite result was obtained after interfering with the expression of C1QBP (Figure 4B). This finding suggests that the *TRIM*5 or *TNFSF*10 gene may mediate the promoting effect of C1QBP on apoptosis. To investigate this, we knocked down the expression of C1QBP followed by treatment with TRIM5 or TNFSF10 to observe whether knockdown of TRIM5 or TNFSF10 could promote apoptosis through the low expression of TRIM5 or TNFSF10. Specific siRNAs (TRIM5-siRNA1, TRIM5-siRNA2, TRIM5-siRNA3, and negative control) knocked down the expression of TRIM5, with TRIM5-siRNA2 reduced the relative expression of TRIM5 mRNA by ~70.49% (*p* < 0.01; Figure 4C), which is significantly more than TRIM5-siRNA1 (no interference efficiency) and TRIM5-siRNA3 (There is no interference efficiency). Thus, TRIM5-siRNA2 was used to silence TRIM5 expression in GFTCs. We examined the cell apoptosis responses to transfection with TRIM5-siRNA2 or negative control, TRIM5 knockdown cells were more prone to apoptosis than those expressing negative control. Meanwhile, the results showed that the apoptosis rate was significantly increased in the TRIM5-siRNA2 and C1QBP-siRNA2 group compared to the only C1QBP-siRNA2 group (Figure 4D). These findings suggest that the expression of TRIM5 regulates C1QBP and thus influences the apoptosis of GFTCs. Like TRIM5, TNFSF10-siRNA2 reduced the relative expression of TNFSF10 mRNA by ~74.348% (*p* < 0.01; Figure 5A), which was used to silence TNFSF10 expression in GFTCs. Meanwhile, we demonstrated no change in apoptosis rate in the TNFSF10-siRNA2 group compared to the negative control. Interestingly, the apoptosis rate was significantly increased in the TNFSF10-siRNA2 plus C1QBP-siRNA2 group compared to the only C1QBP-siRNA2 group (Figure 5B). These results suggest that the expression of TNFSF10 does not affect the apoptosis rate of GFTCs, but the expression of TNFSF10 may can regulate C1QBP, thereby affecting the apoptosis of GFTCs.

## 4 Discussion

Due to ORFV's unique immune mechanism, current vaccines cannot provide complete immune protection to goats. In addition, the existing treatment options for Orf are primarily palliative, as they provide symptomatic treatment and are not able to fundamentally cure the disease. The difficult-to-cure properties of Orf and the recurring outbreaks have become the bottleneck constraining the development of the goat industry. Uncovering the molecular mechanisms of ORFV infection will contribute to developing drugs against Orf, cultivating vaccine candidates, and then managing the current situation in which Orf is only treated symptomatically. Our previous study found that ORFV129 binding to C1QBP and C1QBP can affect ORFV replication. However, the specific mechanism by which C1QBP regulates ORFV replication remains unclear and there currently needs to be a report on the sequence of the goat *C*1*QBP* gene, its protein, and its role in regulating GFTCs. Here, our results revealed that C1QBP negatively regulates cell proliferation while positively regulating apoptosis in GFTCs. What happened can be controlled by both TNFSF10 and TRIM5 expression.

Currently, studies on C1QBP are focused on humans and mice (28–30), and there is no study on the goat *C*1*QBP* gene. Based on the cloned goat *C*1*QBP* gene sequence, we investigate the role of C1QBP on cell proliferation utilizing gene over expression and RNA silencing. Our results showed that silencing of C1QBP can lead to cell proliferation in GFTCs. Our results are also consistent with previous reports in which down regulation of C1QBP was observed to increase cell proliferation in renal carcinoma cell 786-0 (31). The increased C1QBP expression significantly reduced PK-15 cell proliferation (32). On the other hand, as demonstrated in our present study, over expression of the C1QBP protein triggered the opposite implication by inhibiting cell viability in GFTCs. Likewise, over expression of C1QBP has been reported to decrease cell proliferation in SkBr3 cells (33). A study reported that C1QBP silencing induces G1 to S phase arrest in prostate cancer cells (34). In our study, silencing of C1QBP induces the G1 to S phase progression in GFTCs, meaning that cells are progressing to a proliferative stage. At the same time, it was C1QBP over expression led to the opposite result in GFTCs. This finding suggests that C1QBP influences the cell cycle of GFTCs. Cell cycle progression was regulated by the expression of cyclin-dependent kinase (CDK) and cyclin-dependent kinase inhibitors (CK) (35). It has been found that over expression of C1QBP can regulate the transcription of many critical cyclin genes, including cyclin A, photosphere-like kinase 1 (PLK1), cyclin B1, cyclin B2, cyclin-dependent kinase 1 (CDK1), and CDC20 (36). Sensor of cellular mitosis, cyclins, along with their dependent kinases, will collectively regulate the cell cycle progression when DNA damage occurs. This regulation results a G1 phase arrest was observed in cells and facilitates the transition of cells from G1 to S phase, which is conducive to DNA replication (37). In our study, silencing of C1QBP results in the cells to be arrested at the GO/G1 phase, positively affecting cell proliferative. This finding suggests that the change in C1QBP expression might regulate the expression of cellular CDK and cell cycle progression.

Apoptosis is a typical procedural death pathway that can ensure the normal growth and development of the body by eliminating damaged or redundant cells. In our present study, over expression of C1QBP can promote the apoptosis of GFTCs. It has been demonstrated that C1QBP silencing can reduce apoptosis, and this confirmed the function of the C1QBP gene in the opposite direction, which is consistent with Ma, Itahana, and others (38, 39). Apoptosis involves internal (mitochondria) and external (death receptor pathways). Caspase family genes ultimately regulated apoptosis progression, although the signaling pathways were different, and many proteins were involved in regulating apoptosis, for example, Bcl-2, Caspase, and p53 (25, 40, 41). In our study, silencing of C1QBP can significantly alleviate the expression of gene *Caspase*3, *Caspase*7, *Bax, PARP*1, *BCL*2*L*11, and *p*53 genes and increase the expression of *Bcl*-2. This phenomenon was consistent with the change in apoptosis and thus inhibited apoptosis. In the previous study, the Bcl-2/Bax ratio was considered as a molecular switch that can regulate apoptosis. When the Bcl-2/Bax ratio decreases, Bax is more likely to form homodimers, thereby inducing apoptosis. On the other hand, Bcl-2 inhibits apoptosis when the Bcl-2/Bax ratio increases (42). In our study, the Bcl-2/Bax ratio increased relatively when silencing C1QBP, evidence that silencing C1QBP can inhibit apoptosis. Meanwhile, there is no apparent change in Bcl-2 expression after over expression of C1QBP. However, the mRNA expression of Bax relatively increased, and the Bcl-2/Bax ratio decreased, confirming that over expression of C1QBP can promote apoptosis.

The present study analyzed the potential mechanism of C1QBP to regulate apoptosis using RNA-seq. Among these 236 differentially expressed genes, we used correlation screens between DEGs to analyze genes that may play critical roles in regulating apoptosis. The results showed that the essential genes may be YWHAB, TNFSF10, TRIM5, VGF, and PARP1 etc. Apoptosis is a process characterized by chromatin condensation, nuclear shrinkage and apoptosis body production. In the process of apoptosis, numerous signal transduction systems play a pivotal role in delivering apoptotic signals and triggering cell death. During this series of complex reactions, various proteins are involved in regulating the apoptotic signaling pathway, jointly maintaining the balance between cell survival and death. For example, Bcl-2, Caspases, p53, BCL2L11, PARP1, and Caspase-3 serves as the ultimate executor, and its activation can promote cell death (40). The Bcl-2 family (Bcl-2, BCL2L11) regulates cell apoptosis by inhibiting the release of cytochrome C from mitochondria (43). Research demonstrated that C1QBP knockdown reduced the recruitment of the anti-apoptotic proteins, including Bcl-2 and Bcl-XL, and repressed Caspase3 activation and poly (ADP-ribose) polymerase cleavage, which consequently accelerated the T cell apoptotic process (44). C1QBP promotes the catabolism of hypoxanthine and elevates the apoptosis of renal cell carcinoma cells by modulating xanthine dehydrogenase (XDH)-mediated oxygen species (ROS) generation (25). This indicated that there was a connection between the function of Bcl-2, Caspase3, Bax and the functionality of C1QBP. Knockdown of C1QBP can inhibit the proliferation of human embryonic stem cells and promote their apoptosis (45). MiR-128-3p promoted chicken granulosa cell apoptosis through 14-3-3β/FoxO pathway via repressing YWHAB (46). YWHAB inhibits PCV2-induced endoplasmic reticulum stress (ERS), autophagy, reactive oxygen species (ROS) production and apoptosis (47). Aan-miR-181b-3p can induce sensitive cell apoptosis by regulating the expression of TNFSF10, thereby inhibiting Anguillid herpesvirus 1 replication (48). TRIM5 can induce apoptosis in human hepatocellular carcinoma cells by inhibiting the expression level of Bcl-2 and activating the expression of Caspases3 (49). IFIT2 can enhance the apoptotic effect of curcumin-induced leukemia cells (50). This study we demonstrated C1QBP promotes apoptosis in goat turbinate bone cells and showed the effect of C1QBP on the expression of some apoptosis-related genes. Therefore, based on the result of RNA-seq analysis, we further speculate that C1QBP may generally interact with more apoptosis-related proteins, thereby regulating the cell apoptosis.

Go enrichment analysis showed that DEGs were enriched by the response to the virus, activation of the innate immune response, negative regulation of the viral genome, and defense response to the virus, indicating that C1QBP is closely associated with virus infection. And in the previous study, we have already proven that C1QBP has a direct impact on the proliferation of ORFV (9). However, there are still gaps in its role in regulating ORFV infection, and its exploration is necessary for future research. The juncture between cell death and proliferation is a general target for viral infections because viruses need to hinder apoptosis to exploit cells for their own replication (51). ORFV infection can induce cell apoptosis (26) and we have established that C1QBP can promoted apoptosis in goat turbinate bone cells in our study. Therefore, we speculate that C1QBP may play an important role in the cell apoptosis induced by ORFV infection and will conduct experiment to verify this in the further. KEGG enrichment analysis of DEGs showed significant enrichment of Herpes simplex virus 1 infection, Pathways in cancer, PI3K-Akt signaling pathway, Apoptosis, Apoptosis-multiple species and p53 signaling pathway, suggesting that C1QBP is involved in apoptosis. Previous studies have found that stable knockdown of the *C*1*QBP* gene has also been previously shown to inhibit apoptosis in hepatocellular carcinoma cells (52). It was also demonstrated that C1QBP induces apoptosis in cervical cancer cells via the p53-dependent mitochondrial damage pathway (53). However, the specific mechanism of C1QBP in regulating apoptosis is still unclear, and there are no studies on the role of goat C1QBP in regulating apoptosis. Based on the results of RNA-seq analysis, we selected several proteins that may be involved in the regulation of apoptosis and whose functions are unclear. TRIM5 and TNFSF10 are two of them to investigate the role of C1QBP in regulating apoptosis. The TRIM5 protein family has more than 70 members in the human body and is an evolutionarily conservative family involved in many physiological and pathological processes, such as apoptosis and antiviral infections (54, 55). However, to date, the functions of most members of the TRIM5 family are unknown. Our study showed that TRIM5 interference promoted the apoptosis of goat turbinate bone cells and the apoptosis rate was significantly increased in the TRIM5-siRNA2 and C1QBP-siRNA2 groups compared to the only C1QBP-siRNA2 group. These results suggest that the expression of TRIM5 regulates C1QBP and thus influences the apoptosis of GFTCs. TNFSF10 is a tumor necrosis factor ligand super family member that can activate apoptosis in many tumor cell lines (56). EFLDO was identified to sensitize HepG2 cells to TNF super family member 10 (TNFSF10)-induced apoptosis (57). This study showed that compared with the C1QBP-siRNA2 group, the apoptosis rate of the TNFSF10-siRNA2 group plus C1QBP-siRNA2 group was significantly increased, but there was no significant change in the TNFSF10-siRNA2 group compared with the Negative Control group. These results suggest that the expression of TNFSF10 does not affect the apoptosis rate of GFTCs, but the expression of TNFSF10 may can regulate C1QBP, thereby affecting the apoptosis of GFTCs. In summary, we have confirmed that the expression of TRIM5 or TNFSF10 regulates C1QBP and thus influences the apoptosis of GFTCs. And, whether there is a direct interaction between TRIM5 and C1QBP or TNFSF10 and C1QBP remains unclear. We will conduct experiment such as yeast two-hybrid technology and co-immunoprecipitation (Co-IP) assays to verify this in the further.

## 5 Conclusions

Our current work demonstrates that C1QBP plays a significant role in maintaining metabolic activities and that its depletion leads to increased cell viability, cells remaining in the G0/G1 phase, and reduced apoptosis. Conversely, the opposite effect was observed when C1QBP was over expressed in GFTCs. Furthermore, we demonstrated that the expression of TRIM5 or TNFSF10 could regulate the role of C1QBP in apoptosis. This work provides an understanding of the role of C1QBP in apoptosis and could pave the way for further study investigating the role and mechanism of C1QBP protein in mediating the regulation of cell apoptosis by ORFV.

## Data Availability

Raw data have been deposited to National Center for Biotechnology Information (NCBI) under the BioProject number PRJNA1251641.
