# Supplementary material for: C1QBP promotes apoptosis of goat fetal turbinate cells via inhibiting the expression of TRIM5 or TNFSF10
Source: Front Vet Sci. 2025 May 16;12:1524734. doi: 10.3389/fvets.2025.1524734 (PMC12122759; doi:10.3389/fvets.2025.1524734)
Supplement: Supplementary file 1 [file Data_Sheet_1.doc]

*Supplementary Materials*

# **Effect of C1QBP on the apoptosis cycle and proliferation of goat turbinate bone cells**


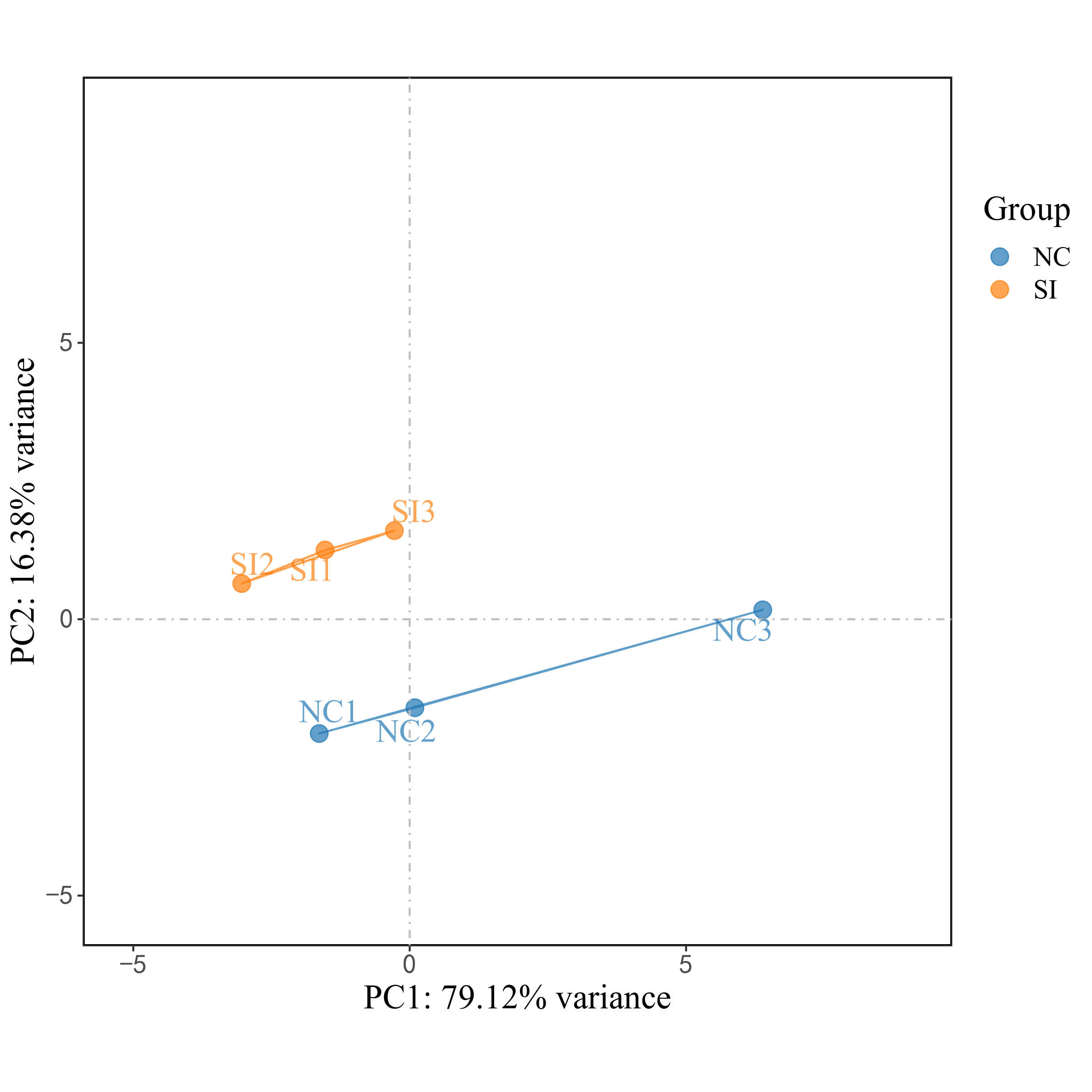


**Figure S1.** Principal component analysis diagram


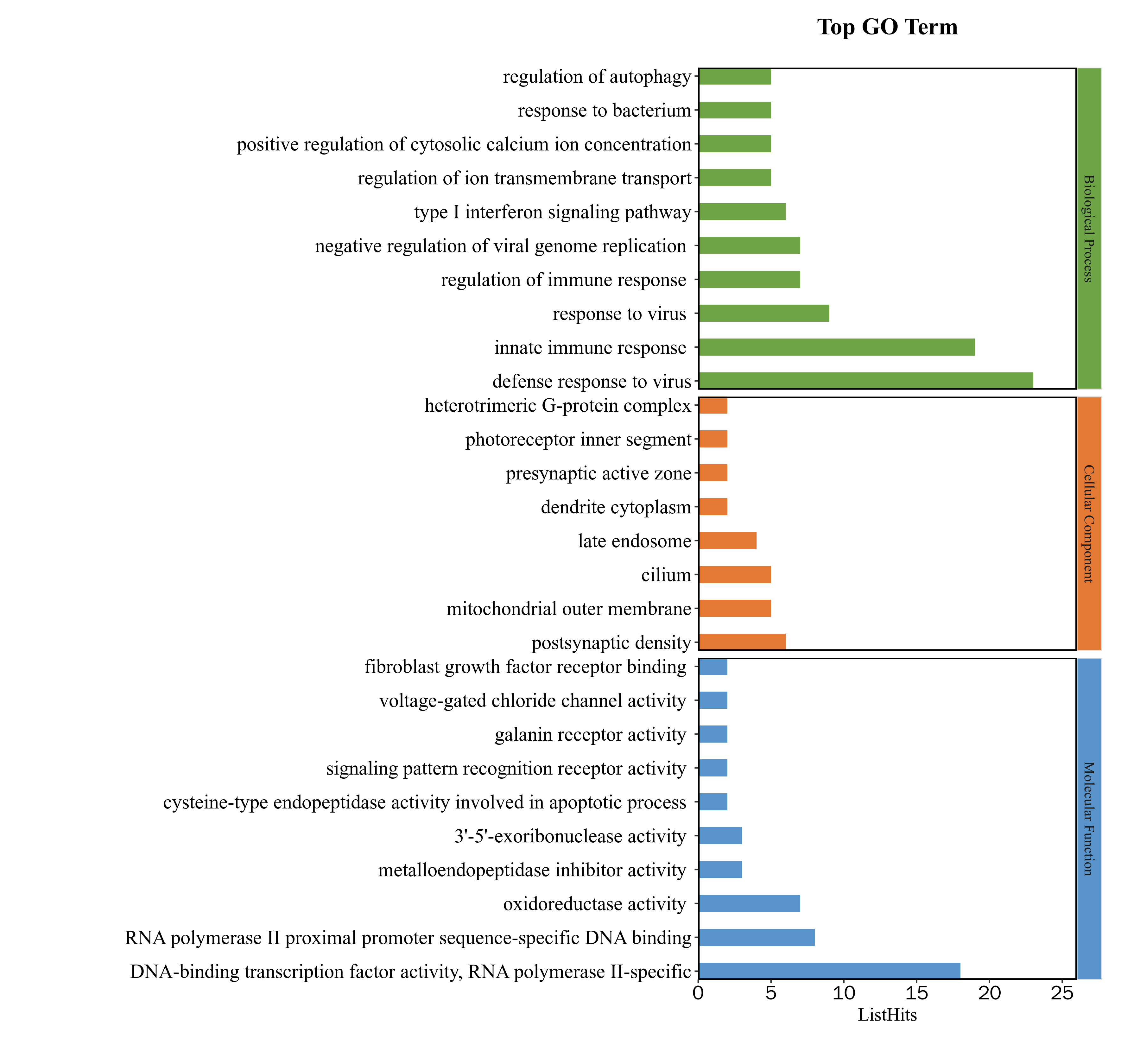


**Figure S2.**Top 30 GO enrichment analysis of DEGs.
